# Individualized sequencing of targeted immunotherapies in generalized myasthenia gravis across distinct patient profiles

**DOI:** 10.3389/fneur.2026.1989043

**Published:** 2026-10-16

**Authors:** Vasiliki Zouvelou, Dimitra Tzavella, Eleni Strataki, Aigli G. Vakrakou

**Affiliations:** First Department of Neurology, Eginition Hospital, School of Medicine, National and Kapodistrian University of Athens, Athens, Greece

**Keywords:** bridging therapy, corticosteroid dose reduction, efgartigimod alfa, generalized myasthenia gravis, ravulizumab, rituximab, rozanolixizumab, targeted immunotherapy

## Abstract

**Background:**

Targeted immunotherapies have expanded treatment options for acetylcholine receptor antibody-positive generalized myasthenia gravis (gMG), but evidence regarding their sequencing and individualized therapeutic pathways remains limited.

**Methods:**

We describe seven selected adults with AChR-positive gMG managed through individualized targeted-treatment pathways involving administered and, in some cases, planned sequential therapy. Treatment selection was guided by disease severity, corticosteroid-related risks, presence of thymoma, comorbidities, peripheral venous access and the need for rapid clinical improvement and/or sustained disease management.

**Results:**

Across the seven cases, FcRn antagonists were used for rapid symptom control, corticosteroid dose reduction and bridging therapy while the appropriate vaccinations required before planned complement inhibition or B-cell depletion therapy were completed. Rituximab was selected as a long-term B-cell-directed immunosuppressive strategy, particularly in recent-onset gMG. Ravulizumab was planned as a long-term, non-steroidal immunosuppressive therapy in thymoma-associated myasthenia gravis and in chronic gMG with symptoms refractory to conventional non-steroidal immunosuppressive therapy and dependent on corticosteroids.

**Conclusion:**

These seven cases illustrate how targeted immunotherapies may be used sequentially and individualized according to their onset of action, MG subgroup, treatment goal, required vaccinations and route of administration.

## 1 Introduction

Generalized myasthenia gravis (gMG) is an antibody-mediated disorder of the neuromuscular junction, most commonly caused by antibodies against the acetylcholine receptor (AChR). Corticosteroids remain the mainstay of treatment, but prolonged exposure is associated with substantial metabolic, cardiovascular, ocular, infectious and skeletal complications. Conventional steroid-sparing immunosuppressants require several months to achieve clinical benefit, creating a need for rapidly acting treatments that can facilitate early corticosteroid reduction and disease control.

The therapeutic landscape of AChR antibody-positive gMG has changed substantially with the introduction of novel targeted immunotherapies. The neonatal Fc receptor (FcRn) antagonists, efgartigimod alfa and rozanolixizumab that promote the degradation of circulating pathogenic AChR antibodies have rapid onset of action and favorable safety profile (1, 2). Ravulizumab, a long-acting terminal C5 complement inhibitor, acts also downstream in the pathogenetic pathway and prevents the formation of Membrane Attack Complex and the complement-mediated injury at the postsynaptic membrane. The CHAMPION MG trial confirmed rapid and lasting improvement in AChR antibody-positive gMG, with maintenance treatment administered every eight weeks (3). Rituximab acts upstream by depleting CD20-positive B cells and may provide long-term immunosuppressive effect. In the RINOMAX trial, rituximab administration within 12 months from diagnosis, increased the probability of achieving minimal disease manifestations and reduced the requirement for rescue treatment compared with placebo (4).

Although targeted therapies are increasingly incorporated into gMG care, evidence remains limited regarding their comparative positioning, optimal sequencing, transition strategies, and patient-specific selection. In routine clinical practice, factors such as the need for rapid symptom control, corticosteroid-related comorbidities, advanced age, thymoma, coexisting malignancy, peripheral venous access and vaccination requirements may influence treatment selection and sequencing.

In this Perspective, we use seven real-world clinical scenarios to illustrate how targeted therapies may be sequenced according to distinct patient profiles and therapeutic objectives, with particular emphasis on rapidly acting treatment strategies, corticosteroid dose reduction, bridging strategies, and transition to sustained therapy.

## 2 Clinical framework and real-world scenarios

### 2.1 Patient selection/source of clinical observations

This retrospective descriptive case series included seven selected adults with AChR-positive gMG managed at the First Department of Neurology, Eginition Hospital, between February 2025 and July 2026. The cases were purposively selected from a broader clinical population to illustrate distinct real-world treatment scenarios. They do not represent a consecutive cohort. Administered targeted therapies are distinguished from subsequent therapies that were planned but had not been initiated by the data cutoff.

Recent-onset gMG was defined as gMG diagnosed within the preceding 12 months (4). Very-late-onset MG was defined as onset at age ≥65 years (5, 6).

### 2.2 Therapeutic considerations

Treatment selection and sequencing were determined by the treating neurologist (VZ) according to disease phenotype and severity, corticosteroid-related risks, presence of thymoma, comorbidities, peripheral venous access, vaccination requirements, response to previous therapies and the need for rapid or sustained disease control. Treatment intervals were individualized according to the clinical needs and the approved dosing schedule of each agent. During the study period, efgartigimod alfa and rozanolixizumab and ravulizumab were the only FcRn antagonists and C5 complement inhibitor, respectively, commercially available in Greece.

### 2.3 Data collection and outcomes

Demographic and clinical data were retrospectively extracted from the patients’ medical records. These included age, sex, disease duration, clinical phenotype, Myasthenia Gravis Foundation of America (MGFA) classification when available, thymoma status, relevant comorbidities, previous and concomitant therapies, targeted-treatment sequence, treatment rationale, corticosteroid dose, clinical response, treatment-related adverse events, and duration of follow-up. MG Activities of Daily Living (MG-ADL) and Quantitative Myasthenia Gravis (QMG) scores were recorded when available.

## 3 Individualized targeted-treatment profiles

### 3.1 Recent-onset gMG: rapid clinical improvement with corticosteroid dose reduction before B-cell-directed therapy

#### 3.1.1 Case 1

A 72-year-old man with a 4-month history of AChR-positive gMG presented with eyelid ptosis, diplopia, dysphagia, dysarthria and axial muscle weakness (MGFA class IIIB). Initial treatment consisted of pyridostigmine 180 mg/day and prednisolone 15 mg/day. His medical history was notable for advanced chronic glaucoma with established visual-field deficits and pathological findings on optical coherence tomography. Because corticosteroid escalation was contraindicated and early corticosteroid reduction was clinically necessary, efgartigimod alfa was initiated as a rapidly acting bridging therapy, with the aim of facilitating corticosteroid dose reduction.

He received four cycles of efgartigimod, administered at intercycle intervals of 5, 4, and 7 weeks, respectively, with clinical stability maintained throughout treatment. During efgartigimod treatment, prednisolone was reduced from 15 to 7.5 mg/day. Rituximab is planned as the longer-term non-steroidal immunosuppressive strategy, based on the RINOMAX trial.

#### 3.1.2 Case 4

A 71-year-old man with recent-onset, AChR-positive gMG of one month’s duration presented with an ocular–bulbar phenotype characterized by eyelid ptosis, dysarthria, dysphagia, nasal speech, and facial weakness (MGFA IIIB). His medical history was notable for recently diagnosed, early-stage prostate cancer (Gleason score 6, clinical stage I–II), for which no oncological treatment had yet been initiated, and arterial hypertension. He was receiving prednisolone 15 mg/day and pyridostigmine 90 mg/day. Given his advanced age and comorbidities, escalation to high-dose corticosteroid therapy was considered inappropriate. Rozanolixizumab was initiated to achieve rapid symptom control and facilitate corticosteroid reduction. He received two rozanolixizumab cycles, separated by an interval of approximately 5 weeks, according to clinical need. The subcutaneous route of administration was also clinically practical. During rozanolixizumab treatment, prednisolone was reduced from 15 to 12.5 mg/day.

Rituximab has not yet been administered but is planned as the longer-term non-steroidal immunosuppressive strategy, based on the RINOMAX trial. Thus, rozanolixizumab is being used as a rapidly acting bridging therapy, with corticosteroid dose reduction during treatment, while the patient remains clinically stable and completes the required vaccinations before rituximab initiation.

#### 3.1.3 Case 5

A 59-year-old man with recent-onset AChR-positive gMG presented with a 1-month history of ocular–cervical–bulbar weakness, including fluctuating ptosis, hoarseness, dysphagia, and neck flexor weakness (MGFA class IIIB). He required hospitalization and received intravenous immunoglobulin, with a favorable but transient response. He was discharged on prednisolone 20 mg/day and pyridostigmine 60 mg/day. His medical history included type 2 diabetes mellitus, arterial hypertension, sinus tachycardia, and gastroesophageal reflux disease, all of which were worsened by corticosteroid therapy.

Because early corticosteroid reduction was clinically necessary, efgartigimod alfa was initiated as a rapidly acting bridging treatment, with the aim of facilitating corticosteroid dose reduction. He received one efgartigimod alfa treatment cycle, with good clinical control and an MG-ADL score of 0 at the latest assessment. Prednisolone was reduced from 20 mg/day to 17.5 mg/day.

Rituximab is planned as the longer-term non-steroidal immunosuppressive strategy, based on the RINOMAX trial. Thus, efgartigimod is used to achieve early clinical improvement and corticosteroid reduction while the patient remained clinically stable and preparation for rituximab therapy with appropriate vaccinations, is completed.

### 3.2 Very-late-onset gMG: continuing FcRn inhibition after partial response to rituximab

#### 3.2.1 Case 2

An 80-year-old man with a 2-month history of very-late-onset AChR-positive gMG, presented with predominant bulbar dysfunction requiring nasogastric tube placement (MGFA IVB). Initial treatment included prednisolone, intravenous immunoglobulin and two rituximab infusions 500 mg each −15 days apart. He achieved clinical improvement, allowing removal of the nasogastric tube; however, dysarthria and dysphagia persisted requiring dietary modifications (MGFA IIIB). Rozanolixizumab was initiated 9 weeks after the second rituximab infusion to provide additional control of the residual bulbar symptoms. Its subcutaneous route of administration was particularly suitable because of the patient’s poor peripheral venous access.

The patient received four rozanolixizumab cycles, with intercycle intervals of 6, 4, and 3 weeks, respectively, determined according to clinical need. Following treatment, his clinical status improved to MGFA class IIB, with milder residual symptoms, while prednisolone was reduced from 10 mg/day at rozanolixizumab initiation to 7.5 mg/day at the latest follow-up.

Before rituximab, MG-ADL was 11, QMG was 14, and MGFA was IVB. Before the first rozanolixizumab infusion, MG-ADL was 6, QMG was 10, MGFA was IIIB, and serum IgG was 763 mg/dL. Peripheral white blood cell count was 7,710/μL and lymphocytes were 31.9% (absolute count 2,459/μL). After each rozanolixizumab cycle, MG-ADL was 3, QMG was 4, and MGFA was IIB. Immunophenotyping 6 months after the last rituximab infusion showed CD19 + cells 0.5% (9 cells/μL).

### 3.3 Thymoma-associated gMG: FcRn bridging to sustained complement inhibition

#### 3.3.1 Case 3

A 46-year-old woman with thymoma-associated, AChR-positive gMG (thymoma AB WHO, IIB Masaoka) was referred to our center approximately 18 months after diagnosis, during which she had not received any treatment for myasthenia gravis. At presentation, she had severe ocular–cervical–bulbar weakness (MGFA IVB).

She was treated with prednisolone, courses of intravenous immunoglobulin and plasma exchange, followed by four weekly rituximab infusions of 500 mg each. These interventions resulted in partial clinical improvement; however, disabling cervical–bulbar deficits persisted, including profound neck flexor weakness, nasal speech, dysarthria, and moderate oral dysphagia (MGFA IIIB).

Efgartigimod alfa was initiated 11 months after the rituximab course as a rapidly acting treatment for the persistent symptoms, with the aim of facilitating corticosteroid dose reduction. She received three efgartigimod cycles, with 7-week intervals between cycles, according to clinical need. Prednisolone was reduced from 20 mg/day at efgartigimod initiation to 10 mg/day at the latest follow-up.

IgG was 785 mg/dL before the first efgartigimod infusion, absolute lymphocyte count was 2,280/μL, and immunophenotyping 15 months after the last rituximab infusion showed CD19 + cells 0.2% (2 cells/μL). No relevant infection history was reported.

Ravulizumab is intended as long-term targeted immunotherapy after completion of the required vaccinations, supported by growing real-world evidence of great response of thymoma-associated gMG to complement inhibitors.

#### 3.3.2 Case 6

A 48-year-old woman with thymoma-associated AChR-positive gMG (thymoma B1 WHO, IIA Masaoka) presented with severe cervical–brachial–bulbar weakness (MGFA IVB). The thymoma had been detected incidentally and surgically removed while gMG developed approximately 2 months after thymectomy. Her medical history was also notable for HPV-associated cervical adenocarcinoma treated with total hysterectomy.

She received prednisolone 25 mg/day and three sessions of plasma exchange, with a favorable clinical response. However, poor peripheral venous access precluded further plasma-exchange sessions. Efgartigimod alfa was therefore introduced as a rapidly acting bridging treatment, while the required vaccinations for future complement inhibition were completed.

She received two efgartigimod cycles, separated by an interval of 4 weeks, with retreatment determined according to clinical need. Her clinical status improved from MGFA IVB to IIA and MG-ADL was 1 (Minimal Symptom Expression) at the latest assessment. Prednisolone remained at 25 mg/day. Baseline IgG was 1,210 mg/dL. Absolute lymphocyte count was 1,600/μL. B-cell immunophenotyping was not available. No relevant infection history was reported.

Ravulizumab has not yet been initiated but is planned as long-term immunotherapy after completion of the required vaccinations. The selection of ravulizumab is supported by growing real-world evidence of great response of thymoma-associated gMG to complement inhibitors.

### 3.4 Chronic corticosteroid-dependent gMG: rapid FcRn-mediated clinical improvement followed by sustained complement inhibition

#### 3.4.1 Case 7

A 71-year-old man with a 7-year history of late-onset AChR-positive gMG, had initially presented with ocular, bulbar, and cervical involvement. He subsequently achieved pharmacological remission with combined treatment with mycophenolate mofetil and prednisolone. While he was on mycophenolate mofetil 1,500 mg/day and prednisolone 5 mg on alternate days, he developed an unprovoked, predominantly ocular–bulbar relapse, characterized by severe bilateral eyelid ptosis and diplopia and mild-to-moderate bulbar involvement, mainly fatigable mastication. Prednisolone was increased to 25 mg/day. Symptom recurrence during corticosteroid tapering indicated inadequate disease control despite mycophenolate mofetil. Efgartigimod-alfa was therefore introduced to achieve clinical improvement of corticosteroid dependent symptoms. He received three efgartigimod cycles. The second cycle was administered after an interval of 10 weeks, whereas the third was given 4 weeks later according to clinical need. Because he was considered no longer responder to mycophenolate mofetil, its dose was reduced from 1,500 to 1,250 mg/day with the intention of gradual discontinuation.

After three efgartigimod cycles, oculobulbar symptoms improved; however, residual ocular manifestations, predominantly ptosis with less prominent diplopia, persisted and tended to recur as the subsequent cycle approached, precluding corticosteroid dose reduction.

Before the first infusion of the first efgartigimod cycle, MG-ADL was 7, QMG was 9, and MGFA class was IIIB. At the post-FcRn assessment, performed 1 week after completion of the therapeutic cycle, MG-ADL was 2, QMG was 3, and MGFA was IIB. Prednisolone remained at 25 mg/day.

Ravulizumab is planned as long-term immunotherapy to provide sustained disease control, given the recurrent, corticosteroid-dependent oculo-bulbar symptoms with prominent ocular involvement. This choice is further supported by a *post hoc* analysis of CHAMPION-MG, which showed benefit across individual MG-ADL items, with particularly notable improvements in ocular symptoms, including eyelid ptosis and diplopia.

The clinical characteristics, treatment sequences, and therapeutic rationale of all patients are summarized in Tables 1, 2. Follow-up duration under the targeted treatment strategy was 7, 12, 5, 4, 1, 3, and 6 months for Cases 1–7, respectively. No treatment-related side effects were recorded in the available data, no relevant infections were identified, and no patient required rescue therapy for worsening gMG during follow-up.

**Table 1:** Individualized targeted-treatment pathways and administered/planned targeted therapies in AChR-positive generalized myasthenia gravis.

Case	MG phenotype	Main clinical need	Therapeutic rationale	Targeted therapy: administered/planned
1	Recent-onset gMG with ocular–bulbar and axial involvement	Avoid corticosteroid escalation because of advanced glaucoma	Rapid clinical improvement and corticosteroid dose reduction while bridging to longer-term B-cell-directed therapy	Administered: efgartigimod alfa. Planned: rituximab.
2	Very-late-onset gMG with persistent bulbar involvement	Ongoing control of residual bulbar symptoms after partial response to rituximab; poor venous access	Continuing, symptom-guided FcRn inhibition as long-term therapy, with the practical advantage of subcutaneous administration	Administered: rituximab → rozanolixizumab.
3	Thymoma-associated gMG with persistent cervical–bulbar deficits	Rapid stabilization and corticosteroid reduction before sustained maintenance therapy	FcRn inhibition as bridging therapy during preparation for planned long-term complement inhibition	Administered: rituximab → efgartigimod alfa. Planned: ravulizumab.
4	Recent-onset gMG with ocular–bulbar involvement	Avoid high-dose corticosteroids because of advanced age and comorbidities	Rapid clinical improvement and corticosteroid dose reduction while preparing for longer-term B-cell-directed therapy	Administered: rozanolixizumab. Planned: rituximab.
5	Recent-onset gMG with ocular–cervical–bulbar involvement	Reduce corticosteroid exposure because of metabolic and cardiovascular comorbidities	Early clinical improvement and corticosteroid dose reduction while preparing for longer-term B-cell-directed therapy	Administered: efgartigimod alfa. Planned: rituximab.
6	Thymoma-associated gMG with severe cervical–brachial–bulbar involvement	Rapid control and bridging during meningococcal vaccination	Immediately available FcRn antagonist before planned sustained complement inhibition	Administered: efgartigimod alfa. Planned: ravulizumab.
7	Chronic late-onset gMG with a predominantly ocular–bulbar relapse	Control corticosteroid-dependent relapse after inadequate response to mycophenolate mofetil	Rapid control of prominent ocular symptoms while preparing for sustained maintenance therapy	Administered: efgartigimod alfa. Planned: ravulizumab.

AChR, acetylcholine receptor; FcRn, neonatal Fc receptor; gMG, generalized myasthenia gravis; IVIG, intravenous immunoglobulin; MG-ADL, Myasthenia Gravis Activities of Daily Living; MGFA, Myasthenia Gravis Foundation of America; PLEX, plasma exchange.

**Table 2:** Patient-level clinical outcomes and follow-up during FcRn-antagonist treatment.

Case	Duration of follow-up (months)	No. of FcRn treatment cycles	Intercycle intervals (weeks)	Pre-FcRn MG-ADL/QMG/MGFA	Post-FcRn MG-ADL/QMG/MGFA	Prednisolone initial → last (mg/day)	Side effects
1	7	4	4–7	0*/2/IIA	0/0/0**	15 → 7.5	No
2	12	4	3–6	6/10/IIIB	3/4/IIB	10 → 7.5	No
3	5	3	7	4/11/IIIB	0/4/IIB	20 → 10	No
4	4	2	5	2/3/IIB	0/0/0	15 → 12.5	No
5	1	1	N/A	2/8/IIIB	0/3/IIA	20 → 17.5	No
6	3	2	4	11/15/IVB	1/5/IIA	25 → 25	No
7	6	3	4–10	7/9/IIIB^†^	2/3/IIB	25 → 25	No

*Case 1: residual weakness before efgartigimod was fatigable axial muscle weakness, which is not captured by the MG-ADL.

**MGFA 0 indicates absence of clinical signs; subsequent classifications follow the standard MGFA ordinal classification (I–V).

MG-ADL, QMG and MGFA before FcRn: before the first infusion of the first therapeutic cycle.

MG-ADL, QMG and MGFA post FcRn: 1 week after completion of the therapeutic cycle.

^†^Case 7: MGFA IIIB was ocular-predominant; this illustrates the inadequacy of the MGFA classification when ocular symptoms are predominant in the context of generalized disease.

Duration of follow-up refers to the duration of follow-up under the targeted treatment discussed.

FcRn, neonatal Fc receptor; MG-ADL, Myasthenia Gravis Activities of Daily Living; MGFA, Myasthenia Gravis Foundation of America; QMG, Quantitative Myasthenia Gravis.

## 4 Discussion

The therapeutic landscape of AChR-positive generalized myasthenia gravis is rapidly evolving, with targeted treatments acting at different levels of the pathogenic cascade. Rituximab acts upstream through B-cell depletion, FcRn antagonists and complement inhibitors act downstream by reducing circulating pathogenic IgG and preventing complement-mediated injury at the neuromuscular junction, respectively. This expanding therapeutic armamentarium creates new opportunities for individualized treatment but also presents an important clinical challenge: identifying which patient is most likely to benefit from each strategy. In the absence of validated predictive biomarkers, treatment selection currently relies largely on clinical characteristics, including disease phenotype and severity, corticosteroid-related risks, presence of thymoma, comorbidities, previous treatment response, route of administration, vaccination requirements. We present seven real-world clinical scenarios illustrating how targeted immunotherapies may be sequenced according to disease phenotype, therapeutic objective, comorbidities, vaccination requirements, and route of administration.

The seven selected cases illustrate four clinically relevant treatment profiles encountered in routine practice. In thymoma-associated gMG (cases 3 and 6), FcRn antagonists were used for rapid clinical improvement and as bridging therapy during the vaccination period before planned initiation of ravulizumab, which was selected for sustained disease control because of its fixed dosing schedule and continuous complement inhibition. Recent real-world evidence supports complement inhibition in thymoma-associated gMG (7, 8). An Italian ravulizumab cohort included 14 patients with thymoma and found a greater early QMG response in this subgroup, while a separate cohort of 23 patients supported the effectiveness of complement inhibition in thymoma-associated disease (9, 10).

In gMG of recent onset (cases 1,4,5), FcRn antagonists provided rapid clinical improvement, corticosteroid reduction when feasible and bridging while preparing for rituximab. For Cases 1, 4, and 5, rituximab is planned as a single 500-mg IV infusion, based on the RINOMAX regimen (4) and additional evidence supporting rituximab in recent-onset gMG (11–13). In our practice, rituximab is planned at least 2–3 weeks after the last FcRn-antagonist dose and after recovery of serum IgG levels, preferably to >5 g/L. Limiting prolonged corticosteroid exposure was particularly relevant in Case 1 because of advanced glaucoma, in Case 4 because of age and comorbidity, and in Case 5 because of steroid-related metabolic comorbidity. The planned 2–3-week interval reflects efgartigimod pharmacokinetics and published MG sequencing experience (14, 15). In our practice, this interval is used together with serum IgG assessment before rituximab. Νo evidence-based interval between FcRn inhibition and rituximab has been established.

In very-late-onset MG (case 2) with persistent bulbar symptoms and partial response to rituximab, rozanolixizumab was used as a continuing long-term treatment with symptom-guided cyclic administration and the practical advantage of the subcutaneous route. Because FcRn inhibition enhances IgG catabolism and rituximab is an IgG1 monoclonal antibody, a pharmacokinetic interaction is possible. Νo evidence-based interval between FcRn inhibition and rituximab has been established. Finally, in relapsing, corticosteroid-dependent gMG with prominent ocular involvement (case 7) and inadequate response to mycophenolate mofetil, efgartigimod was used for rapid symptoms control while ravulizumab was planned as sustained maintenance; this rationale was additionally supported by a *post hoc* CHAMPION-MG analysis suggesting benefit in ocular MG-ADL items (16).

Clinically meaningful improvement with efgartigimod may be observed as early as 1 week after the first administration, with maximal improvement generally occurring around Week 4, whereas improvement with rozanolixizumab has been detected from Day 8, the first post-baseline assessment (1, 17, 18). Emerging real-world evidence supports the use of FcRn for corticosteroid tapering, individualized retreatment and bridging before thymectomy or initiation of longer-term immunosuppression (19). However, FcRn antagonists may also be used as an individualized continuing treatment strategy rather than exclusively as bridging therapy.

In the very-late-onset MG (Case 2), rozanolixizumab was initiated 9 weeks after the second rituximab infusion to control persistent bulbar symptoms despite a partial response to rituximab. This timing was clinically determined and should not be interpreted as a validated pharmacokinetic interval. Accordingly, the 9-week interval used in our patient reflects individualized clinical practice rather than a recommended washout period.

An important practical consideration when planning C5 complement inhibition therapy is the requirement for meningococcal vaccination. Vaccination against serogroups A, C, W, Y, and B should be completed or updated at least 2 weeks before the first ravulizumab dose. When treatment must begin sooner, appropriate antibacterial prophylaxis is required until 2 weeks after vaccination (20). In contrast, FcRn antagonists do not require specific pre-treatment vaccinations, allowing treatment to be initiated without the vaccination delay associated with C5 inhibition (1,2). In our patients, FcRn antagonists were therefore used as immediately available bridging therapies, during the vaccination period before planned ravulizumab initiation (cases 3,6,7).

The treatment pathways were sequential rather than concomitant. Several planned transitions had not yet been implemented at the data cutoff. Sequential treatment assigns distinct clinical roles to each agent: rapid reduction of circulating pathogenic IgG with an FcRn antagonist, long-term B-cell-directed immunosuppression with rituximab and sustained terminal complement inhibition with ravulizumab. Evidence for true concomitant FcRn and C5 inhibition remains limited. Irie et al. recently reported two patients with refractory AChR-positive gMG treated with combined blockade of both pathways using subcutaneous efgartigimod and zilucoplan, a macrocyclic peptide C5 inhibitor (21). Because zilucoplan is not an IgG monoclonal antibody, it is not expected to be affected by FcRn-mediated IgG catabolism. By contrast, ravulizumab is an IgG-based monoclonal antibody, and FcRn blockade increases its clearance. No validated washout interval between efgartigimod or rozanolixizumab and ravulizumab has been established. Zilucoplan was not commercially available in Greece during the study period and was therefore not a treatment option in our clinical practice.

Limitations include the retrospective single-center design, very small and heterogeneous sample, potential selection bias, physician-directed treatment selection, incomplete standardized longitudinal assessments, concomitant therapies, variable follow-up duration, and the fact that several proposed treatment transitions had not yet been implemented. The selected cases are therefore descriptive and hypothesis-generating and do not establish comparative efficacy or an optimal treatment sequence.

Overall, these real-world clinical scenarios illustrate how targeted treatments may be assigned distinct but complementary roles according to patient profile and therapeutic objective. FcRn antagonists were used as rapidly acting therapies that, depending on the clinical context, served as either bridging or continuing treatment, with corticosteroid dose reductions documented in several cases. Rituximab was selected as a longer-term strategy, particularly in recent-onset disease, and ravulizumab was selected as a sustained-maintenance strategy in thymoma-associated and recurrent, corticosteroid-dependent gMG. Rather than defining a fixed treatment algorithm, these observations highlight the potential value of tailoring treatment sequence to disease phenotype, therapeutic objective, comorbidities, vaccination requirements, and route of administration. Prospective studies are needed to establish comparative efficacy, safety, optimal treatment intervals, and biomarkers capable of guiding individualized treatment selection.

## Data Availability

The original contributions presented in the study are included in the article/supplementary material, further inquiries can be directed to the corresponding author.
